# Public Health Interventions for the COVID-19 Pandemic Reduce Respiratory Tract Infection-Related Visits at Pediatric Emergency Departments in Taiwan

**DOI:** 10.3389/fpubh.2020.604089

**Published:** 2020-12-16

**Authors:** Chien-Fu Lin, Ying-Hsien Huang, Chi-Yung Cheng, Kuan-Han Wu, Kuo-Shu Tang, I-Min Chiu

**Affiliations:** ^1^Department of Emergency Medicine, Kaohsiung Chang Gung Memorial Hospital, Kaohsiung, Taiwan; ^2^Department of Pediatrics, Kaohsiung Chang Gung Memorial Hospital, Kaohsiung, Taiwan; ^3^Chang Gung University College of Medicine, Taoyuan, Taiwan; ^4^Department of Computer Science and Engineering, National Sun Yet-Sen University, Kaohsiung, Taiwan

**Keywords:** public health interventions, social distancing, wearing masks, handwashing, respiratory tract infection, COVID-19, pediatric emergency department, SARS-CoV-2

## Abstract

**Background and objective:** Public health interventions such as social distancing, wearing surgical or N95 masks, and handwashing are effective in significantly reducing the risk of infection. The purpose of this article is to analyze the effect of public health interventions on respiratory tract infection-related visits to pediatric emergency departments during the COVID-19 pandemic in Taiwan.

**Method:** Pediatric emergency department visits between January 1 2020 and April 30 2020 were included for trend analysis and compared to the same period during the past 3 years. The datasets were retrieved from Taiwan National Infectious Disease Statistics System and Kaohsiung Chang Gung Memorial Hospital. Respiratory tract infections with other diagnoses categories, including fever, asthma, and urinary tract infections, were included for subgroup analysis.

**Result:** A significant decrease of more than 50% in respiratory tract infection-related visits was found from February to April 2020 in the national database. With regard to diagnosis category, the proportion of respiratory tract infections in Kaohsiung Chang Gung Hospital also became significantly lower in 2020 during the months of March (43.4 vs. 37.4%, *p* = 0.024) and April (40.1 vs. 32.2%, *p* < 0.001). On the other hand, the proportion of urinary tract infections was significantly higher in 2020 during March (3.7 vs. 5.2%, *p* = 0.033) and April (3.9 vs. 6.5%, *p* < 0.001), and that of asthma was also higher in April (1.6 vs. 2.6%, *p* = 0.025). Furthermore, the intensive care unit admission rate was relatively higher in 2020 from February, with significant differences noted in March (1.3 vs. 2.8%, *p* < 0.001).

**Conclusion:** Due to public health interventions for the COVID-19 pandemic, the transmission of not only COVID-19 but also other air droplet transmitted diseases in children may have been effectively prevented.

## Introduction

Coronavirus Disease 2019 (COVID-19) has emerged as a new infection (1, 2) and began infiltrating modern society from the beginning of 2020 (3, 4). Its rapid local and international spread, in addition to its ability to infect large numbers of crowds, including health care professionals, some of whom required intensive care, has generated considerable media attention (5). Emergency departments (EDs) around the world have been flooded by patients suspected of having the coronavirus infection. However, while frontline health care workers have been exhausted handling COVID-19 patients, ED visits related to non-coronavirus illnesses fell drastically during the same period. This scenario reflects the same situation in 2004, where studies from that time demonstrated a decrease of up to 50% of ED visits during the SARS epidemic (6, 7). Parents may have been avoiding the hospital because it presents the greatest risk of exposure to the virus, raising concerns about the aerosolized spread of the virus by being exposed to other coughing respiratory patients and aerosol-generating procedures (8, 9). Furthermore, parents may view the hospital as a risky location and are unaware of the cleaning precautions or screening methods that have been adopted (10). This decline was interpreted as “COVID-phobia,” where patients were assumed to be avoiding hospitals due to a fear of contracting the COVID-19 infection while visiting the ED. However, public health interventions for the COVID-19 pandemic also play an important role in non-coronavirus illnesses. Steps for epidemic control, including wearing face masks, washing hands, and social distancing, have been well-executed among the general worldwide population.

During the COVID-19 pandemic, Taiwan has so far been able to protect the interests of its citizens thanks to rapid alerts and by following emergency management activation (11, 12). We believe that such measures not only inhibited the transmission of the COVID-19 infection but also other contagious diseases (13), especially for the pediatric population, where infectious diseases account for 80% of ED visits (14). One study has shown that maintaining social distance and wearing surgical or N95 masks are both effective methods for significantly reducing the risk of infection as droplets are generated at the face level, which made masks crucial for protection during the SARS epidemic (15). Furthermore, handwashing has long been regarded as a vital and cost-effective infection-control practice against the transmission of SARS and other respiratory contagious diseases in health care and community settings (16). Therefore, the aims of this study are to analyze the effect of public health interventions on respiratory tract infection (RTI)-related visits to pediatric EDs during the COVID-19 pandemic in Taiwan.

## Methods

### Patient Population

In this study, we first included the dataset from Taiwan's National Infectious Disease Statistics System on the Taiwan Centers for Disease Control public website for trend analysis regarding RTI-related visits nationally (17). And we further analyze disease specific pediatric ED visits from the electric medical records of Kaohsiung Chang Gung Memorial Hospital, one of the largest medical centers in southern Taiwan, to address the effect of public health intervention on common pediatric ED diagnosis. In this study, pediatric ED visits during January 1 2020 and April 30 2020, were included for trend analysis and were compared with the same period during the past 3 years. We established the end of January as the turning point for distinguishing the changing number of ED visits based on the timeline of COVID-19 in Taiwan, in which the first confirmed case of COVID-19 was diagnosed on January 21 2020 and face mask rationing began February 6 2020 (18).

### Data Collection

Outcome variables included for comparison were total number of pediatric ED visits, rate change of common pediatric ED diseases, and patient's disposition during studied period. Common pediatric infectious diseases include fever and RTI-related visits were enrolled for analysis. And although no apparent literature has elaborated on the direct relationship between epidemic prevention policies and conditions like asthma and urinary tract infection (UTI), we have observed that trends of these two disease diagnosis demonstrated no reduction during the last pandemic period when personal protective measures were adopted to reduce pandemic influenza transmission in 2009 in the US (19–21). Therefore, we also included asthma and UTI as outcome variables and hypothesized that these diseases' prevalence should be less associated with epidemic prevention policies.

Data of RTI-related visits were directly retrieved from database of Taiwan Centers for Disease Control public website. And for disease associated specific ED visit, we used the International Classification of Diseases, 10th revision (ICD-10) codes to retrieve included diseases directly from studied hospital's administrative database. We defined fever as R509, UTI as N390 and asthma as J4520-J45998. RTI-related visits were collected from ICD-10 codes R05 (cough), J00-J219 (respiratory tract infection) and were confirmed by history and image study. Non-existent or unutilized ICD-10 codes between the above diagnosis categories were skipped or abandoned after carefully examining all details in the electronic charts. Patients' disposition were collected as secondary outcomes, including pediatric intensive unit admission and general ward admission were considered for further analysis.

### Statistical Analysis

All values in the figures and tables are expressed as mean ± standard deviation. Quantitative data were analyzed using the *t*-test or one-way analysis of variance with tukey's HSD test as *post hoc* test when appropriate. Two-sided *p*-values < 0.05 were considered statistically significant. All statistical analyses were performed using SPSS statistical software (SPSS for MAC, version 22; SPSS). All patients' records and information were anonymized and de-identified prior to review and analysis. The institutional review board of the Chang Gung Medical Foundation approved this study (IRB number: 202000840B0).

## Results

### RTI-Related Pediatric ED Visits During January to April From 2017 to 2020 in Taiwan

**Figure 1:**
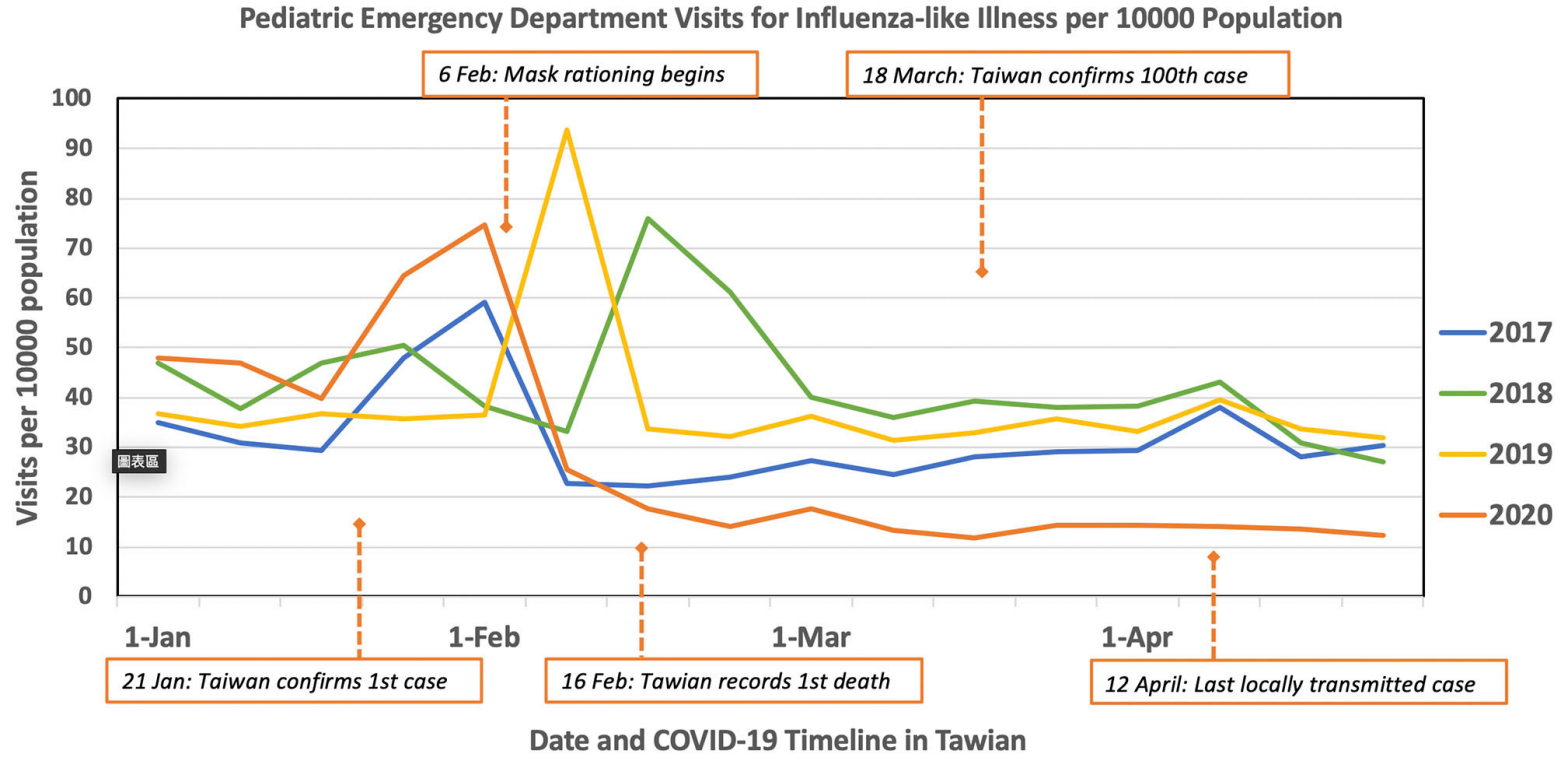
Comparison of respiration tract related pediatric ED visit per 10,000 population between 2017, 2018, 2019, and 2020 in Taiwan.

First, we observed cases of RTI-related visits drop by approximately 50%, from 49.9 to 25.7 per 10,000 people, since the 2nd week in February, compared to the average over the past 3 years (Figure 1) in the nation-wide database (22). In March and April, the decreased patient numbers were more obvious compared to previous years (March: 33.3–14.3, April: 32.9–13.6 per 10,000 people).

### Daily Pediatric ED Visits During January to April From 2017 to 2020 in Studied Hospital

**Figure 2:**
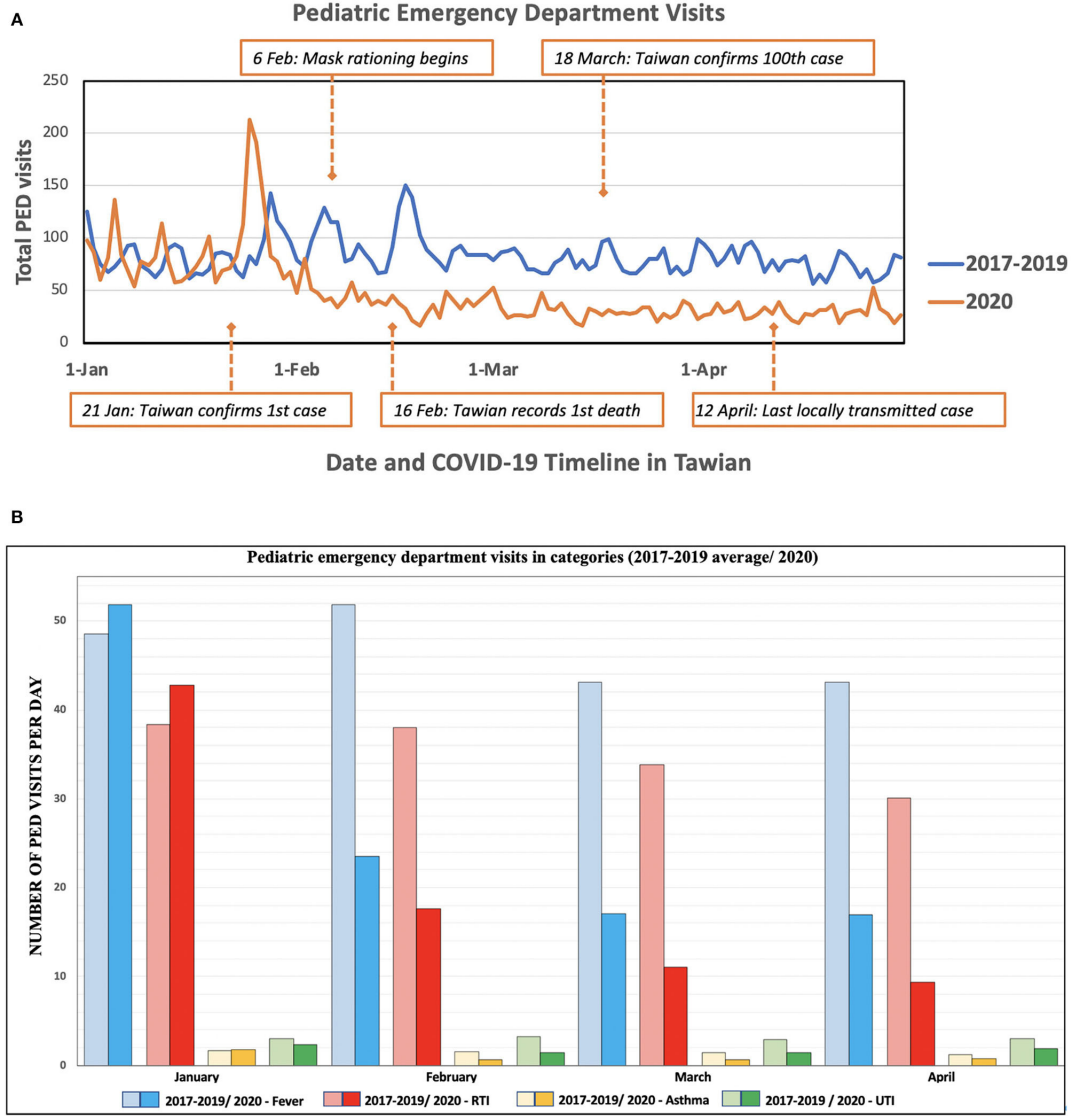
Comparison of average visits from 2017 to 2019 and for 2020 at Kaohsiung Chang Gung Memorial Hospital. **(A)** Total visits at pediatric emergency department. **(B)** Pediatric emergency department visits in categories. PED, pediatric emergency department; RTI, respiratory tract infection; UTI, Urinary tract infection.

We compared the trends of pediatric ED visits in studied Hospital, including number of visits and ratio over the past 3 years. Table 1 shows the average ED visits per day during January to April from 2017 to 2020 by month. At studied Hospital, we also observed statistically significant differences between group averages during February to April (all *p* < 0.001), but not in January (*p* = 0.097) in ANOVA analysis. Apart from 2020, ED visits among 2017, 2018, and 2019 appear to be homogenous. After *post hoc* test applied, daily ED visits of 2017 were higher than 2019 (94 ± 40.5 vs. 73 ± 16.5, *p* = 0.042) in January. On February, ED visits in 2020 were significantly lower than other 3 years (41 ± 11.4, *p* < 0.001), while visits in 2017 were also lower than visits in 2018 (73 ± 18.0 vs. 109 ± 62.1, *p* = 0.017). Afterward, daily ED visits of 2020 were both lower than other 3 years in March (30 ± 7.2, *p* < 0.001) and April (29 ± 7.7, *p* < 0.001). The result is displayed in Table 1. We also demonstrated the number of ED visits per day in specific diagnosis categories from 2017 to 2020 presented in mean value and noted that all of the diagnosis-related visits dropped significantly from February 2020 (Figure 2).

**Table 1:** Comparison of ED visits per day during the period of January to April from 2017–2020 at Kaohsiung Chang Gung Memorial Hospital.

	**January**		**February**		**March**		**April**	
	**Mean** **±** **SD**	***p*****-value**	**Mean** **±** **SD**	***p*****-value**	**Mean** **±** **SD**	***p*****-value**	**Mean** **±** **SD**	***p*****-value**
**2017**	94 ± 40.5^a^	0.097	73 ± 18.0^c^	**<0.001**	72 ± 16.2	**<0.001**	73 ± 21.7	**<0.001**
**2018**	87 ± 23.9		109 ± 62.1		85 ± 20.4		77 ± 19.7	
**2019**	73 ± 16.5^a^		100 ± 46.4		78 ± 23.0		78 ± 17.0	
**2020**	88 ± 36.3		41 ± 11.4^b^		30 ± 7.2^*^		29 ± 7.7^*^	

a daily ED visits of 2017 were higher than 2019 (94 ± 40.5 vs. 73 ± 16.5, p = 0.042).

b daily ED visits in 2020 were significantly lower than other 3 years (41 ± 11.4, p < 0.001).

c daily ED visits in 2017 were lower than visits in 2018 (73 ± 18.0 vs. 109 ± 62.1, p = 0.017).

* daily ED visits of 2020 were both lower than other 3 years in March (30 ± 7.2, p < 0.001) and April (29 ± 7.7, p < 0.001).

*Bold text indicates a statistically significant difference with a p-value less than 0.05*.

### Disease Classification During January to April From 2017 to 2020

Regarding changes to ED visits for the selected diagnoses, we found that the proportion of ED visits among diagnoses showed no statistical differences between 2020 and 2017–2019 in January and February (Table 2). As time passed, the proportion of RTI became significantly lower in 2020 in March (43.4% vs. 37.4%, *p* = 0.024) and April (40.1% vs. 32.2%, *p* < 0.001). The proportion of UTI was significantly higher in 2020 in March (3.7% vs. 5.2%, *p* = 0.033) and April (3.9% vs. 6.5%, *p* < 0.001), and that of asthma was also higher in April (1.6% vs. 2.6%, *p* = 0.025). When we look closely into the analysis of the total and ICU admission ratios, the total admission ratio, including the general ward and ICU, demonstrated an elevation trend since March. Notably, the ICU admission rate was relatively higher in 2020 from February with significant differences noted in March (1.3% vs. 2.8%, *p* < 0.001). On the other hand, we observed no statistically significant monthly differences in admission rate between 2020 and the past 3 years.

**Table 2:** Number of ED visits and ratio (%) regarding diagnosis and patient disposition at Kaohsiung Chang Gung Memorial Hospital.

	**January**			**February**			**March**			**April**		
	**17–19**	**2020**	***p*****-value**	**17–19**	**2020**	***p*****-value**	**17–19**	**2020**	***p*****-value**	**17–19**	**2020**	***p*****-value**
**Total ED visits**	7845	2745		7888	1176		7231	921		6869	875	
**Diagnosis**												
Fever	4519 (58.6)	1607 (58.5)	0.658	4343 (55.1)	682 (58.0)	0.315	4010 (55.5)	529 (57.4)	0.545	3876 (56.4)	510 (58.3)	0.584
RTI	3570 (45.5)	1326 (48.3)	0.126	3191 (40.5)	510 (43.4)	0.223	3140 (43.4)	344 (37.4)	***0.024***	2754 (40.1)	282 (32.2)	***<0.001***
Asthma	158 (2.0)	54 (2.0)	0.883	133 (1.7)	17 (1.4)	0.972	135 (1.9)	22 (2.4)	0.288	108 (1.6)	23 (2.6)	***0.025***
UTI	275 (3.5)	74 (2.7)	0.057	277 (3.5)	43 (3.7)	0.809	268 (3.7)	48 (5.2)	***0.033***	268 (3.9)	57 (6.5)	***<0.001***
**Disposition**												
Admission	1638 (20.9)	522 (19.0)	0.089	1432 (18.2)	245 (20.8)	0.069	1634 (22.6)	231 (25.1)	0.184	1623 (23.6)	191 (21.8)	0.348
ICU admission	92 (1.2)	25 (0.9)	0.263	101 (1.3)	22 (1.9)	0.108	95 (1.3)	26 (2.8)	***<0.001***	79 (1.1)	15 (1.7)	0.157

RTI, Respiratory Tract Infection; UTI, Urinary Tract Infection; ICU, Intensive Care Unit.

*Bold text indicates a statistically significant difference with a p-value less than 0.05*.

## Discussion

In this study, we first observed a nationwide trend of RTI-related pediatric ED visit dropping by ~50% after executing mask policies in response to the COVID-19 pandemic, when compared to the average over the past 3 years (Figure 1). Like many other countries in the world, a dramatic decline of ED visits has occurred since the beginning of the pandemic, particularly in February 2020. All these phenomena may be related to a number of reasons. One of the factors may be the so-called COVID-phobia (23) that may make parents anxious about bringing their sick children to the hospital. The relationship between COVID-19-phobia and the decline in patient volume was also observed in our study.

The other factor may be the benefit from the efficient implementation of public health interventions, including wearing face masks, washing hands, and social distancing in Taiwan. Since contagious and infectious diseases make up the great majority of pediatric emergency department cases, similar results were found when we compared the trends of pediatric ED visits. Therefore, we further reviewed visit data from the pediatric emergency department at Kaohsiung Chang Gung Memorial Hospital before and during the COVID-19 pandemic to help differentiate other reasons for the drop in visits. The volume of patients presenting to KCGMH pediatric emergency department dropped significantly by more than 50% among all diagnostic categories since February while struggling with the COVID-19 pandemic. The ratio of ICU admission in February 2020, though not statistically significant compared to the period of 2017–2019, rose from 0.9–1.9% since critically ill patients are urged to seek medical help under life threatening conditions regardless of their fear of contracting a respiratory contagious disease. This trend can be verified by epidemiologic analysis (24) and annual total admission amount calculation (25) during the 2009 H1N1 pandemic in the US.

It is worth mentioning that the ratio of ICU and general ward admissions in January 2020 was lower than in 2017–2019. The main reason is that, according to the lunar calendar, Taiwan was celebrating Chinese New Year in January 2020. The Chinese New Year festival influences patients and their parents' behavior in seeking health care. In Taiwan, the Chinese New Year festival lasts for 1 week. All the local medical clinics are closed during this week, and patients can only seek medical assistance through the ED. This phenomenon results in an increase in non-urgent ED visits during Chinese New Year. Furthermore, the ratio of ICU admissions in March 2020 was much higher than the average. The major reason was that total pediatric ED visit volume significantly decreased, which elevated the ratio of ICU admissions in March 2020. Another hypothesis could be delayed medical treatment due to population panic over the COVID-19 pandemic, as we traced the COVID-19 timeline in Taiwan, recording the first death on 16 February and confirming the 100th case on 18 March (Figure 1). This phenomenon of delayed medical intervention was also observed in the adult emergency department (26). It is understandable that parents have been reluctant to bring their children to a hospital during the COVID-19 outbreak, thus explaining the potential delays in seeking care.

The ratio of asthma, which we classified as a non-contagious disease, was not significantly higher from March, as we expected; the same was true of urinary tract infections. Multiple factors may contribute to this result. First, COVID-19 is a global pandemic and a serious threat to human health that has halted economic activities, thus reducing air pollution and reclaiming nature (27). Young children with asthma are particularly susceptible to air pollution due to their developing lungs, immature metabolic pathways, high breathing rates per bodyweight, and amount of time spent exercising outdoors (28). Therefore, young children may actually benefit from this phenomenon. Furthermore, asthma, though not a contagious disease, may also benefit from wearing masks. It is most likely that the face mask may make it easier for the wearer to maintain a normal body temperature, while the airway mucosa is protected from cooling and dehydration, two factors that may cause an increased incidence of asthma (29).

One of the main results in this study demonstrates that UTI could be considered a “panic phenomenon” that reflexes people's medical attention-seeking mentality. A lower ratio was noted in January and February in 2020 at the beginning of the pandemic. After people in Taiwan regained confidence in the government's pandemic prevention measures (30) and were willing to seek medical advice, we saw the last locally confirmed transmitted case on 12 April (Figures 1, 2). The ratio of UTI, which is classified as a non-contagious disease based on its epidemiology and pathogenic mechanism (31), has been significantly higher since March 2020. The ratios of hospital admission reached a general level in April 2020 after people regained confidence in the government and hospitals' pandemic prevention measures. No further locally transmitted case has been reported since 12 April 2020 in Taiwan, as demonstrated in both Figures 1, 2.

The most important result of our study indicates a robust connection with the government's policies. Due to relevant policies (mask rationing on 6 February in Taiwan) and their mandated and comprehensive execution, not only did RTI-related cases in KCGMH reflect statistical significance, but the trend of RTI in Taiwan also showed obvious declines (32). Our pediatric emergency department experienced a significantly reduced proportion of RTI patients in 2020 in March (37.4%) and April (32.2%) compared to the ratio in January (48.3%). We believe this result may be attributed to the triad of public health interventions in Taiwan (33), which include wearing face masks, washing hands, and social distancing.

Our study has several limitations that should be mentioned at this point. First, it was conducted in a single and pediatric tertiary care emergency department, so the generalizability of these findings is limited to comparable institutions. Second, how the public used the pediatric emergency department during the COVID-19 outbreak may not reflect future use. However, we still found that a lower ratio of respiratory tract infection, with regard to air droplet transmitted diseases, has a correlation with epidemic prevention in both the national database and the studied hospital. Urinary tract infections, defined as a non-contagious disease and “panic phenomenon,” strongly reflect people's medical-seeking mentality.

In conclusion, we believe that the decline in pediatric ED visits may partly come from panic. However, our study has also shown that the triad of public health interventions for the COVID-19 pandemic may have effectively prevented the transmission of not only COVID-19 but also other air droplet transmitted diseases in children.

## Data Availability Statement

The raw data supporting the conclusions of this article will be made available by the authors, without undue reservation.

## Ethics Statement

The institutional review board of the Chang Gung Medical Foundation approved this study (IRB number: 202000840B0).

## Author Contributions

C-FL, I-MC, and Y-HH: conceptualization. C-FL and I-MC: data curation, formal analysis, investigation, methodology, and Writing - original draft. I-MC: Project administration. C-YC, K-HW, and Y-HH: Resources. C-FL, C-YC, and Y-HH: Software. K-HW, Y-HH, K-ST, and I-MC: Supervision. Y-HH and I-MC: Validation. C-FL, I-MC, and Y-HH: Visualization and Writing - review and editing. All authors contributed to the article and approved the submitted version.

## Conflict of Interest

The authors declare that the research was conducted in the absence of any commercial or financial relationships that could be construed as a potential conflict of interest.
